# Femto-SMILE after photo-therapeutic keratectomy in an eye with failed LASIK flap: A case report

**DOI:** 10.1016/j.ajoc.2023.101852

**Published:** 2023-04-29

**Authors:** Ramy Awad, Khaled Awad, Ahmed Bakry, Moones Abdalla

**Affiliations:** a Alexandria General Ophthalmology Hospital, Alexandria, Egypt; b Roayah Vision Correction Center, Alexandria, Egypt

**Keywords:** Flap complications, Topo-PTK, Femto-SMILE, Primary LASIK

## Abstract

**Purpose:**

The aim of the study is to describe a case where Femtosecond Intrastromal Lenticule Extraction was used to address the refractive defect following topography-guided phototherapeutic keratectomy (topo-PTK) to regularise a scarred corneal surface after an initial LASIK flap formation attempt failed.

**Observations:**

A 23-year-old female experienced a thin and irregular corneal flap, during a microkeratome LASIK surgery of the right eye. Subsequently, she experienced epithelial ingrowth. Three months postoperatively the cornea showed scarring and partial flap melting. Topo-PTK was employed to ablate the scarred surface rendering it regular. Treatment with Femtosecond Intrastromal Lenticule Extraction was performed to correct the final refractive error of Sph −5.50 Cyl −2.00 Axis 180° with a happy end of uncorrected visual acuity (UCVA) of 20/20.

**Conclusions and Importance:**

Femtosecond Intrastromal Lenticule Extraction can be used for retreatment after surface ablation. Topo-PTK can be used to ablate post-operative LASIK-induced irregularities with a successful outcome.

## 1 Introduction

In the realm of ophthalmology, laser in situ keratomileusis (LASIK) is one of the most frequently performed procedures. Its widespread use is due to the procedure's effectiveness, safety, quick recovery, and high level of patient comfort.^1^ When utilizing a microkeratome to create a flap, the LASIK technique entitles the incidence of several dangers for the eye, including buttonholes, free caps, thin incomplete or uneven flaps, and postoperative epithelial ingrowth, corneal scarring, or melting.^2^^,^^3^

In the all-laser approach known as Femtosecond Intrastromal Lenticule Extraction, the refractive error is rectified by removing a lenticule rather than using an excimer laser for photo-ablation. This lowers the possibility of problems from a flap.^4^^,^^5^

We describe a case where Femtosecond Intrastromal Lenticule Extraction was used to address the refractive defect following topography-guided phototherapeutic keratectomy (topo-PTK) to regularise a scarred corneal surface after an initial LASIK flap formation attempt failed.

## 2 Case report

A 23-year-old female medical student presented requesting refractive surgery. Her UCVA was 20/400 in both eyes, and manifest refraction in the right eye was Sph −3.50 Cyl −0.50 Axis 40° (BCVA 20/20), and the left eye was Sph −3.50 Cyl −0.25 Axis 160° (BCVA 20/20). The patient had no history of ocular or systemic disease, the anterior and posterior segment examination with a slit-lamp biomicroscope were normal, and the IOP was 16 mmHg in both eyes (using Goldmann ApplanationTonometer “GAT” at 2:00 p.m.). The keratometry values were 43.0 D axis 5°, 44.1 D axis 95° and 42.9 D axis 169°, 43.8 D axis 79° in the right and left eyes respectively. The thinnest points on the corneal pachymetry map were 517 μ and 512 μ in the right and left eyes, respectively. After discussion with the patient, we decided to choose LASIK rather than Photorefractive keratectomy or Femto-second laser-assisted lenticule extraction. The reason for choosing LASIK over Femto-second laser-assisted lenticule extraction was a financial issue related to the patient.

A regular inspection of the M2 Moria microkeratome was done before the procedure. To rule out any blade flaws, the microkeratome was inspected under a microscope. After wetting the disposable blade with a balanced salt solution (BSS), the oscillation was evaluated.

Radial and para-radial corneal markings were created inferotemporal with gentian violet, commencing with the right eye. A suction ring of size “0” with stop “8” was placed on the eye. The microkeratome assembly was positioned and secured after achieving sufficient suction. A few drops of BSS were placed inside the ring and the microkeratome was activated. On removing the suction ring microkeratome assembly, the flap created was thin, irregular, and buttonholed with epithelium covering a large part of the bed. There was also a free cap, and the flap was removed with the spatula. A second inspection revealed no issues with the microkeratome.

Consequently, the procedure in the right eye was halted and laser ablation was not performed. The uneven detached flap was softly replaced, and the corneal markings were realigned. BSS was used to maintain hydration of the flap and cornea. A bandage soft contact lens was applied. After replacing the motor, head, ring, and microkeratome blades on the microkeratome, the procedure for the left eye was carried out without any issues. The laser ablation was performed using the Wavelight EX500 excimer laser platform (Alcon Inc., Fort Worth, Texas, USA).

The patient was apprised of what had happened, the potential for postoperative problems, and the subsequent care actions. Moxifloxacin, Prednisolone acetate, and lubricating eye drops every 3 h were prescribed to the patient before she was discharged with safety netting.

The flap revealed increasing epithelial ingrowth throughout the patient's follow-up at one day, one week, and one month after surgery. Because re-lifting the flap and scraping were not practical and may result in irreversible loss of the flap, conservative therapy was preferred. However, after 6 weeks, the patient developed progressive melting of a part of the flap with scarring of other parts, resulting in an irregular corneal surface with an asymmetric bowtie on Pentacam (Fig. 1a). However, detailed epithelial thickness measurements were not accessible at the time.

**Fig. 1a:**
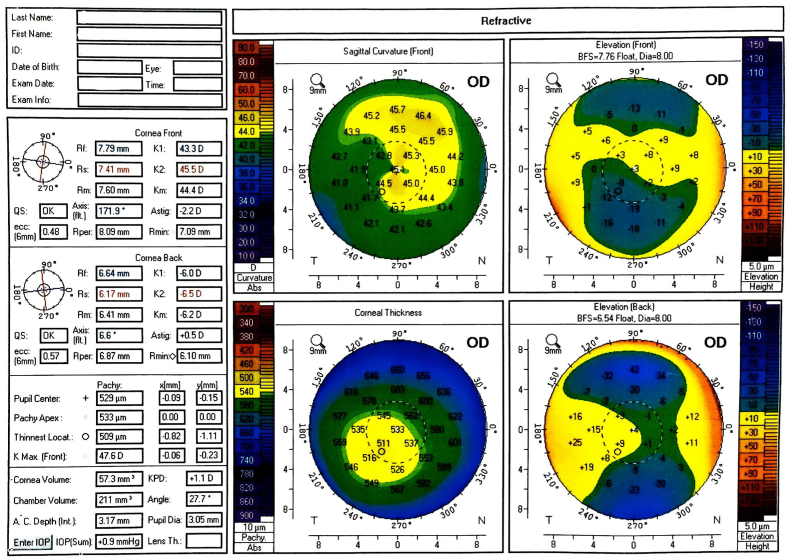
The four refractive maps of Pentacam Topography of the right eye showing irregular corneal surface with asymmetric bowtie.

After discussion with the patient and obtaining informed consent, topo-PTK was performed, while the flap was left in position and not removed manually. We ablated 100 μ (50 μ then 50 μ) using the software of Wavelight EX500 excimer laser platform (Alcon Inc., Fort Worth, Texas, USA). Mitomycin C with a concentration of 0.02% was then applied for 60 seconds after the second step of the procedure. Following the procedure, the corneal surface became more regular (Fig. 1 b).

**Fig. 1b:**
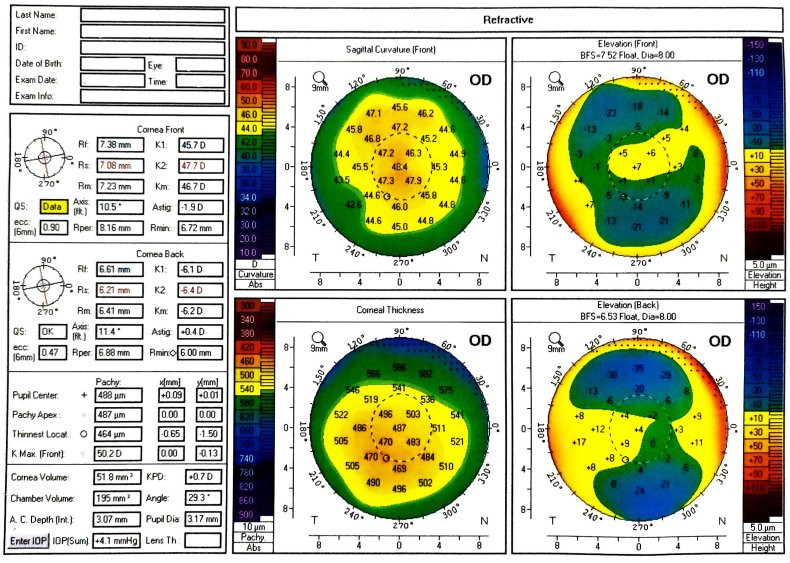
The four refractive maps of Pentacam Topography after Topo-PTK showing more regular corneal surface.

**Fig. 2:**
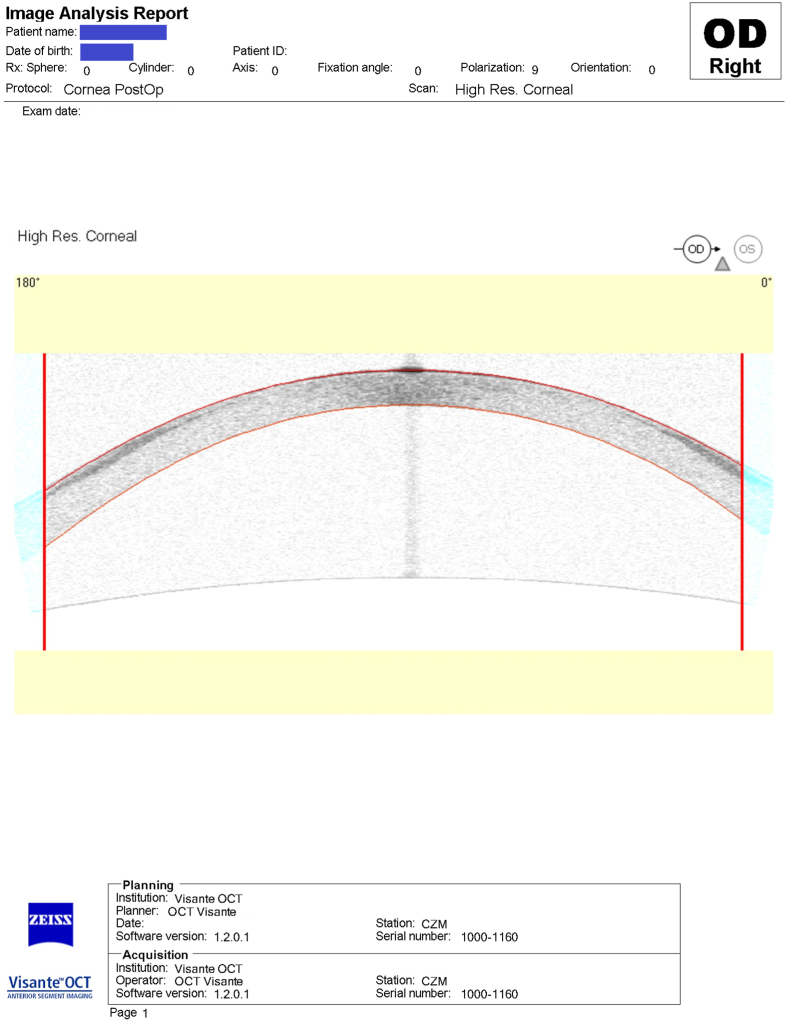
Anterior segment OCT showing midperipheral circumferential corneal scarring following PTK.

The patient acquired a circumferential peripheral scar during a three-month follow-up (Fig. 2). We administered a potent steroid therapy to the scar (Prednisolone acetate 2 hourly). After two weeks of therapy, the scar notably faded. However, the patient complained of some sort of ocular heaviness and one-sided headache. The IOP was 36 mmHg and 13 mmHg in the right and left eyes, respectively “using GAT at 12:45 p.m. with thickness compensation”. We decided to rapidly downgrade steroids over a week and prescribed Dorzolamide-Timolol combination, non-steroidal anti-inflammatory drops “Nepafenac”, four times per day, artificial tears, along with Ascorbic acid 1000 mg tablets, three times per day. We closely monitored the IOP measurement, ocular inflammatory signs, and changes in the corneal scar.

After 3 months, the IOP was normal, the scar was minimal and peripheral, and the refraction was stable Sph −5.50 Cyl −2.00 Axis 180° (BCVA 20/20). the corneal thickness was 468 μ, and the keratometry values were 44.1 D axis 18°, 45.8 D axis 108°. There were four options for correction of such error: contact lens use, Photorefractive Keratectomy (PRK), Femtosecond Intrastromal Lenticule Extraction, or lenticular surgery (Phakic intraocular lens). After discussion with the patient about options, risks, and potential hazards we decided to Proceed with Femtosecond Intrastromal Lenticule Extraction.

**Fig. 3:**
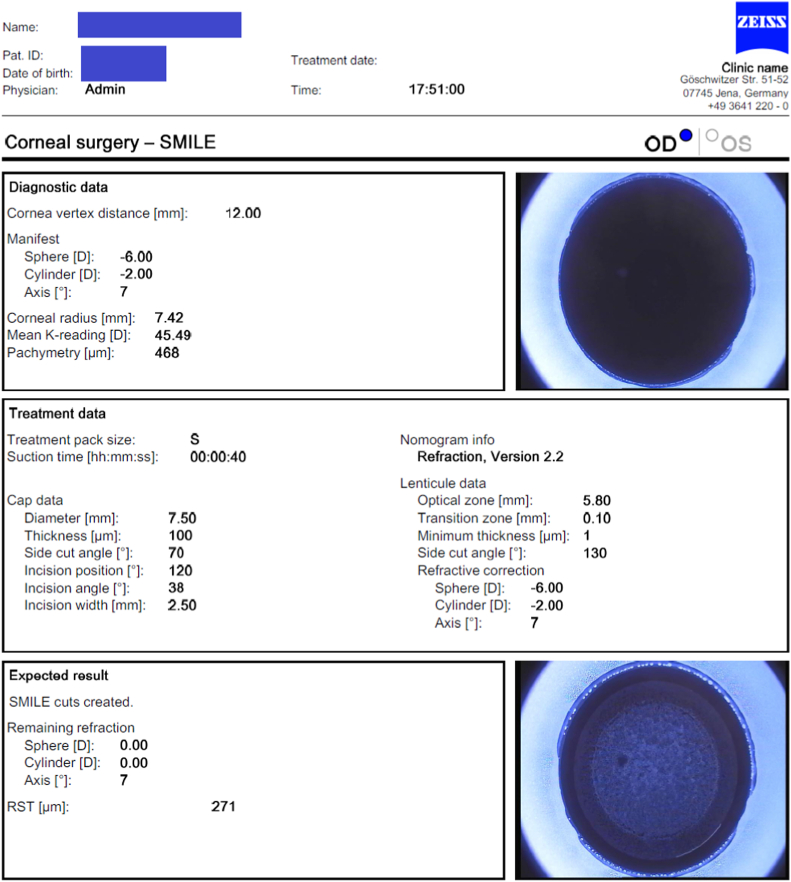
Patient data used for femto-SMILE procedure (Visumax 500 - Carl Zeiss meditec).

With some modifications to the data, Sph −6.0 Cyl −2.0 Axis 7° (Fig. 3), We performed Femtosecond laser-assisted Small Incision Lenticule Extraction (Femto-SMILE). Myopic overcorrection by 0.5 D was intended for fear of postoperative regression. Axis was changed to approach the topographic axis on Pentacam. The procedure was performed using VisuMax® (Carl Zeiss Meditec, Jena, Germany), with a cap diameter of 7.5 mm, a cap thickness of 100 μm, an optical zone of 5.8 mm and a transition zone of 0.10 mm, and an expected residual stromal thickness of 271 μ. The minimum lenticule thickness of 1.0 μm was available in the software of VisuMax 500 before the last update. Manual limbal marking was performed on the slit lamp before the operation for compensation of cyclotorsion and achievement of a good astigmatic correction. The case was found to be challenging during the dissection of the lenticule from the cap beside the presence of a black spot paracentral at the 9 o'clock position, however, the procedure was successful (Refer to the attached Video).

Supplementary video related to this article can be found at doi:10.1016/j.ajoc.2023.101852

The following is/are the supplementary data related to this article:

Multimedia component 1 1 Multimedia component 1

**Fig. 4:**
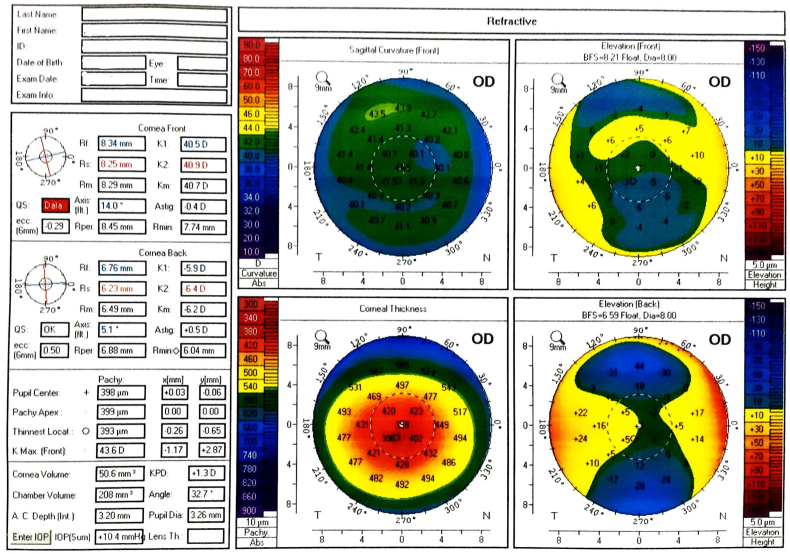
The four refractive maps of Pentacam Topography of the right eye six months after Femto-SMILE.

Follow-up of the patient was at one day, one week, one month, and six months postoperatively. The final UCVA is 20/20 for each eye, the cornea was clear, IOP was normal, the corneal surface was regular (Fig. 4) and the refraction was insignificant. The patient was satisfied without any complaints related to visual quality or optical aberrations.

## 3 Discussion

During LASIK, flap creation using a mechanical microkeratome is a critical step with many risks such as a failed flap that may result from microkeratome failure. With the use of femtosecond lasers for flap creation and the increase in surgical experience, the incidence of such complications has decreased significantly. It was previously estimated to occur in 0.3–1.2% of cases.^6^ Photo-therapeutic keratectomy (PTK) has been described for the treatment of several corneal surface irregularities caused by many conditions including post-LASIK partially amputated flap, as well as corneal opacities caused by post-adenoviral corneal opacities. Topography maps have been shown to be vital for improving the success of PTK procedures by guiding the excimer laser for tailored corneal ablation values.^7^, ^8^

In our case, we experienced failed LASIK flap mostly because of improper suction or loss of suction during flap creation. Flap appeared to be thin, irregular, and buttonholed, which may contribute to the postoperative epithelial ingrowth in which epithelial cells migrate from the buttonhole to the under-surface of the flap. We choose topo-PTK for the treatment of the corneal scar to obtain a regular corneal surface, especially in the presence of partial melting of the flap.

Zhao et al.^9^ described a case of failed LASIK flap for which they performed PTK and an autologous lenticule transplant obtained via Femto-SMILE of the contralateral eye. They reported a clear cornea with a UCVA of 20/22 after two years of follow-up.

Garcia-Gonzalez et al.^10^ Reported a case of flap amputation and PRK with mitomycin C to treat central flap necrosis after a LASIK retreatment. Although they got the final visual acuity of 20/25, their patient still has some corneal irregularity and a very thin cornea.

In our case, we successfully treated the final refractive error using Femtosecond Intrastromal Lenticule Extraction. This is a minimally invasive procedure that involves the formation of a corneal intrastromal lenticule using a femtosecond laser, followed by lenticule removal through a small side cut. It is a safe, effective, and stable procedure for the correction of myopia with good predictability.^11^

We choose Femtosecond Intrastromal Lenticule Extraction over PRK because of its ability to correct higher refractive errors with less risk of corneal haze, especially in our patient with a history of corneal scarring following PTK. Na et al.^12^ Reported an increased incidence of corneal haze following PRK for moderate and high myopia rather than low errors. In their multicenter study, the incidence of postoperative corneal haze was 4.6% for myopia less than 6 D, while in moderate myopia 6–10 D, the incidence increased to 16%.

Femtosecond Intrastromal Lenticule Extraction was also less invasive rather than Phakic intraocular lens (PIOL). Moya et al.^13^ assessed long-term outcomes of PIOL (Implantable Collamer Lens – ICL) in adults and reported the possibility of postoperative cataract formation, pupillary-block glaucoma, and dislocation. Aruma et al.^14^ evaluated the visual outcomes of SMILE versus ICL for moderate myopia and found that both provide good efficacy, safety, and predictability with more visual complaints following ICL.

To the best of our knowledge, no previous studies reported Femtosecond Intrastromal Lenticule Extraction following surface ablation. On the other hand, Leccisotti et al.^15^ investigated the use of femtosecond LASIK for retreatment after PRK and reported excellent refractive outcomes.

The difficulty in the procedure lies in the dissection of a thin weak cap without bowman's membrane which was ablated during PTK. In contrast, Theo Seiler et al.^16^ performed a creep test and stress relaxation to assess corneal viscoelastic properties in the absence of Bowman's membrane. They found that the contribution of the Bowman's membrane to the corneal stability was not significant.

The history of corneal scarring was also the cause of difficulty in dissection besides the appearance of a black spot in the lenticule. Hamed et al.^17^ reported about 1% incidence of black islands in their cases reaching the visual axis. It was associated with difficult dissection of the lenticule but not affecting the outcome of the procedure.

Despite such challenges, careful planning and a well-informed patient decision were instrumental to achieving a successful patient-centered treatment plan with an ideal outcome. Further follow-up of the patient was recommended to ensure stable refraction and exclude the presence of ectasia.

## 4 Conclusions

In conclusion, Femtosecond Intrastromal Lenticule Extraction can be used for retreatment after surface ablation. Topo-PTK can be used to ablate post-operative LASIK-induced irregularities with a successful outcome.

## Patient consent statement

The patient has provided a written informed consent for the publication of this report. The patient signed the consent, after discussion with a research assistant who informed her about the publication and data confidentiality.

## Funding

No funding or grant support.

## Authorship

All authors attest that they meet the current ICMJE criteria for Authorship.

## Declaration of competing interest

All authors (RA, KA, AE, MA) have no financial interests to disclose.
